# Runx1 is a key regulator of articular cartilage homeostasis by orchestrating YAP, TGFβ, and Wnt signaling in articular cartilage formation and osteoarthritis

**DOI:** 10.1038/s41413-022-00231-y

**Published:** 2022-10-28

**Authors:** Yan Zhang, Tao Zuo, Abigail McVicar, Hui-Lin Yang, Yi-Ping Li, Wei Chen

**Affiliations:** 1 Department of Pathology, University of Alabama at Birmingham, Birmingham, AL 35294 USA; 2 Key Laboratory of Biomedical Information Engineering of Ministry of Education, Biomedical Informatics and Genomics Center, School of Life Science and Technology, Xi’an Jiaotong University, Xi’an, 710049 Shaanxi P.R. China; 3 Department of Orthopaedics, the First Affiliated Hospital of Soochow University, Orthopaedic Institute of Soochow University, 899 Pinghai Road, Suzhou, 215031 Jiangsu P.R. China; 4 Division in Cellular and Molecular Medicine, Department of Pathology and Laboratory Medicine, Tulane University School of Medicine, Tulane University, New Orleans, LA 70112 USA

**Keywords:** Bone, Homeostasis

## Abstract

Runt-related transcription factor 1 (Runx1) plays a key role in cartilage formation, but its function in articular cartilage formation is unclear. We generated non-inducible and inducible Runx1-deficient mice (*Runx1*^*f/f*^*Col2α1-Cre* and *Runx1*^*f/f*^*Col2α1-CreER* mice) and found that chondrocyte-specific Runx1-deficient mice developed a spontaneous osteoarthritis (OA)-like phenotype and showed exacerbated articular cartilage destruction under OA, characterized by articular cartilage degradation and cartilage ossification, with decreased Col2α1 expression and increased Mmp13 and Adamts5 expression. RNA-sequencing analysis of hip articular cartilage from the *Runx1*^*f/f*^*Col2α1-Cre* mice compared to that from wild-type mice and subsequent validation analyses demonstrated that Runx1 is a central regulator in multiple signaling pathways, converging signals of the Hippo/Yap, TGFβ/Smad, and Wnt/β-catenin pathways into a complex network to regulate the expression of downstream genes, thereby controlling a series of osteoarthritic pathological processes. RNA-sequencing analysis of mutant knee joints showed that Runx1’s role in signaling pathways in articular cartilage is different from that in whole knee joints, indicating that Runx1 regulation is tissue-specific. Histopathologic analysis confirmed that Runx1 deficiency decreased the levels of YAP and p-Smad2/3 and increased the levels of active β-catenin. Overexpression of Runx1 dramatically increased YAP expression in chondrocytes. Adeno-associated virus-mediated Runx1 overexpression in the knee joints of osteoarthritic mice showed the protective effect of Runx1 on articular cartilage damaged in OA. Our results notably showed that Runx1 is a central regulator of articular cartilage homeostasis by orchestrating the YAP, TGFβ, and Wnt signaling pathways in the formation of articular cartilage and OA, and targeting Runx1 and its downstream genes may facilitate the design of novel therapeutic approaches for OA.

## Introduction

Osteoarthritis (OA) is the most common degenerative joint disease and a major cause of pain and disability that often leads to severely limited mobility and physical disabilities for people over the age of 55 years.^1^ OA is characterized by cartilage degradation, subchondral bone thickening, osteophyte formation, and high expression of the cartilage degradation enzymes MMPs and ADAMTSs.^1–3^ Current treatments for OA are often palliative and in many cases require joint replacements,^2^ which are costly, risky and functionally finite. The root causes of articular cartilage degeneration in OA remain unclear. Thus, a complete understanding of the pathological mechanisms is critical to developing therapies for OA.

Runt-related transcription factor 1 (Runx1) is a key transcription factor in the development of the hematopoietic system that also regulates early chondrocyte formation during bone development and fracture healing.^3–7^ Our previous studies revealed that Runx1 could regulate the BMP/TGFβ/Smad and Wnt/β-catenin signaling pathways and orchestrate multiple signaling pathways in bone, contributing to the earliest stages of skeletogenesis.^8,9^ Moreover, we found that Runx1 attenuated chondrocyte to osteoblast lineage commitment and inhibited bone formation by limiting both chondrogenesis and osteogenesis.^10^ We revealed that Runx1 deficiency in chondrocytes resulted in downregulated chondrocyte hypertrophy gene expression, which delayed chondrocyte differentiation.^10^ Based on previous findings, we suspected that Runx1 may play a role in cartilage repair and OA. In addition, some studies on Runx1 have focused on the early stage of cartilage development and cartilage hypertrophy.^10,11^ A meta-analysis of a genome-wide association study identified some loci related to the shape of the hip and found that Runx1 is involved in hip OA and fractures.^12^ However, the function and mechanisms underlying the role of Runx1 in OA and postnatal articular cartilage regeneration remain unclear.

In this study, to explore the role of Runx1 in articular cartilage, we generated chondrocyte-specific Runx1-deficient mice (*Runx1*^*f/f*^*Col2α1-Cre* mice). We also used the inducible Cre-FloxP system to mediate time-specific gene knockout to produce postnatal *Runx1*^*f/f*^*Col2α1-Cre* ER mice. We found that chondrocyte-specific Runx1-deficient mice developed a spontaneous OA-like phenotype. We performed RNA-sequencing analysis to investigate the mechanism underlying the role of Runx1 in OA and found that Runx1 may orchestrate the YAP, TGFβ, and Wnt signaling pathways and that Runx1 is also involved in multiple biological processes (BP), including the inflammatory response, bone regeneration, biomineral tissue development, collagen fibril organization and tissue development. We further confirmed that Runx1 could significantly promote YAP and p-Smad2/3 expression in chondrocytes and cartilage, which may be the mechanism of the significantly impaired articular cartilage regeneration and repair leading to OA, and Runx1 could inhibit active β-catenin expression by regulating YAP expression, thereby limiting the formation of osteophytes. Furthermore, our study demonstrated that adeno-associated virus (AAV)-mediated local Runx1 overexpression protected against surgical OA in mice. These results indicated that Runx1 could protect articular cartilage from OA and could be a potential drug target in the treatment of OA.

## Results

### Loss of Runx1 in chondrocytes exacerbated articular cartilage damage after DMM surgery

**Fig. 1:**
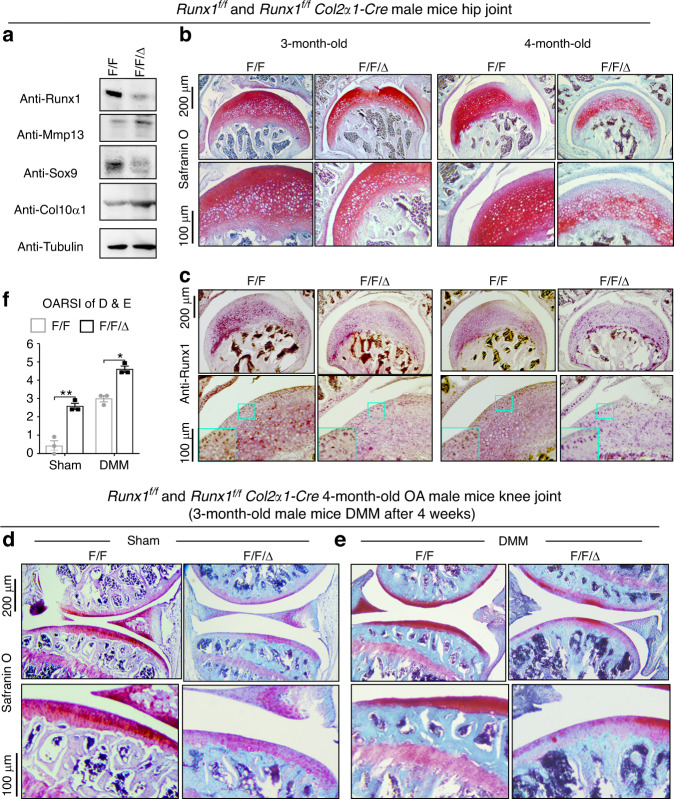
Loss of Runx1 in chondrocytes exacerbated articular cartilage damage after DMM surgery. **a** Western blot of knee joints from 4.5-month-old *Runx1*^*f/f*^ (F/F) and *Runx1*^*f/f*^ *Col2α1-Cre* (F/F/Δ) mice. **b** Safranin O (SO) staining for hip joint. **c** Anti-Runx1 immunohistochemistry (IHC) staining of the hip joint of 3- and 4-month-old mice. **d**, **e** Safranin O (SO) staining of the knee joint of 3-month-old mice after (**d**) sham and (**e**) DMM surgery. **f** Knee joint and hip joint OARSI scores of (**d** and **e**). The results are presented as the mean ± SD, *n* = 3, *P < 0.05, **P < 0.01

To explore the role of Runx1 in articular cartilage, we generated *Runx1*^*f/f*^*Col2α1-Cre* (F/F/Δ) mice by crossing *Runx1*^*f/f*^ (F/F) mice with Col2α1-Cre mice to specifically delete Runx1 expression in chondrocytes. Western blot results showed successful Runx1 knockdown in articular cartilage of the *Runx1*^*f/f*^*Col2α1-Cre* mice, as well as increased protein levels of the articular cartilage damage-related genes Mmp13 and Col10α1 with a decrease in Sox9 expression in the Runx1-deficient mice (Fig. 1a). Moreover, Safranin O (SO) staining showed that loss of Runx1 promoted articular cartilage loss compared to that of the control mice in the hip joints (Fig. 1b). The mutant mice had shorter hypertrophic zones than the littermate controls, which resulted in malformed growth plates in the long bones of the mutant mice. The intensity of SO staining was directly proportional to the proteoglycan content in normal cartilage, and as shown by histology, the mutant mice showed a modest decrease in SO staining intensity, indicating decreased proteoglycan content in the mutant mouse cartilage (Fig. 1b). Moreover, IHC staining results confirmed Runx1 depletion in the cartilage of the *Runx1*^*f/f*^*Col2α1-Cre* (F/F/Δ) mice (Fig. 1c). To examine the effect of Runx1 on OA progression, we performed destabilization of the medial meniscus (DMM) surgery, which can generate a well-established mouse model to mimic human OA.^13^ The Runx1-deficient mice had a significant increase in articular cartilage loss and thus OA severity (Fig. 1d, e). Our results showed that Runx1 deficiency in chondrocytes also led to an increase in the Osteoarthritis Research Society International (OARSI) score compared to that of the sham controls (Fig. 1f). TRAP staining was used to determine potential differences in osteoclasts in the control and mutant mice under physiological and surgically induced osteoarthritic conditions (Fig. S1D, E). Osteoclast numbers were similar between the WT and mutant mice in the sham group, but TRAP^+^ osteoclasts were increased twofold in the mutant mice following DMM surgery (Fig. S1D, E). These data indicated that loss of Runx1 in chondrocytes leads to cartilage damage under physiological conditions and exacerbates articular cartilage defects under osteoarthritic conditions, which suggests that Runx1 may play crucial roles in osteoarthritic cartilage destruction.

### Mice with postnatal Runx1 deletion exhibited a spontaneous OA-like phenotype

**Fig. 2:**
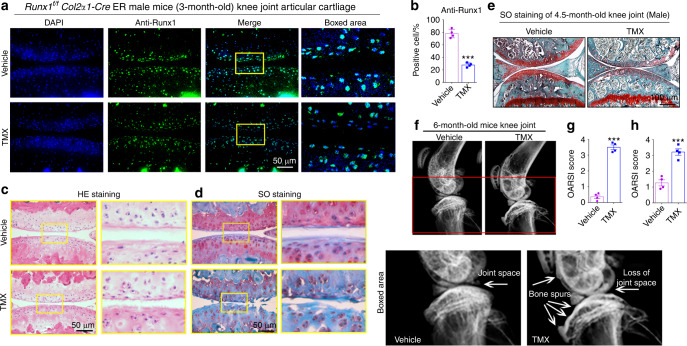
*Runx1*^*f/f*^ *Col2α1-Cre* ER mice showed a spontaneous OA-like phenotype in vivo. **a** IF staining of anti-Runx1 in articular cartilage of 3-month-old male *Runx1*^*f/f*^ *Col2α1-Cre ER* mice induced by vehicle (control) and TMX. Bar: 50 μm. **b** Quantification of (**a**). **c** H&E (HE) staining of 3-month-old male mice. Bar: 50 μm. **d** Safranin O (SO) staining of 3-month-old male mice. Bar: 50 μm. **e** SO staining of 4.5-month-old male mice. Bar: 100 μm. **f** Radiographic image of 6-month-old *Runx1*^*f/f*^ *Col2α1-Cre ER* mice. The white arrow represents the osteophytes and a narrowed joint space. **g**, **h** Knee joint OARSI scores of (**d** and **e**). The results are presented as the mean ± SD, *n* = 4, ***P < 0.001

To exclude the effect of Runx1 on cartilage development and to further investigate the role of Runx1 in cartilage loss, we generated *Runx1*^*f/f*^*Col2α1-Cre ER* mice by crossing *Runx1*^*f/f*^ mice with *Col2α1-Cre ER* mice and injected tamoxifen (TMX) to induce postnatal Runx1 deletion in cartilage. First, we detected the genotype of the *Runx1*^*f/f*^*Col2α1-Cre ER* mice before and after TMX induction and found that the deletion band (298 bp) appeared after induction with TMX (Fig. S1A). We found no significant differences in body length between the 2-month-old vehicle (control) and TMX-induced *Runx1*^*f/f*^*Col2α1-Cre* *ER* mice (Fig. S1B), while examination of the femur and tibia lengths in the 8-month-old WT, vehicle (control) and TMX-induced *Runx1*^*f/f*^*Col2α1-Cre* *ER* mice similarly showed no significant difference in length (Fig. S2). Furthermore, we examined Runx1 expression in the *Runx1*^*f/f*^*Col2α1-Cre* *ER* mice by induction with vehicle and TMX and confirmed that postnatal Runx1 was successfully knocked down in articular cartilage by TMX injection (Fig. 2a, b). In addition, histological bone phenotypes for the knee joint of 3-month-old mice with spontaneous OA showed dysregulated joint space and damaged joint morphology in the postnatal Runx1-deficient mice by H&E and SO staining (Fig. 2c, d, g). Moreover, SO staining of the 4.5-month-old mice with TMX exhibited obvious articular cartilage erosion and closed joint space (Fig. 2e, h). Furthermore, a narrow joint space and osteophytes were found in the knee joint of the TMX-treated mice by radioactive imaging (Fig. 2f). Our data suggested that postnatal Runx1 deficiency in mouse cartilage resulted in a spontaneous OA-like phenotype.

### Postnatal Runx1 deficiency in articular cartilage decreased Col2α1 expression and increased the expression of cartilage degradation markers

**Fig. 3:**
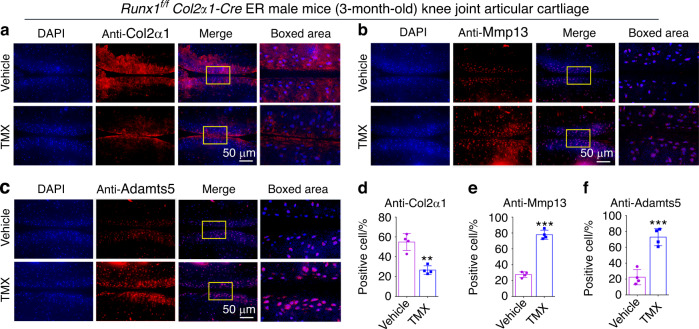
Postnatal Runx1 deficiency in cartilage resulted in decreased Col2α1 expression and increased Mmp13 and Adamts5 expression. **a–c** IF staining with anti-Col2α1 (**a**), anti-MMP13 (**b**) and anti-Adamts5 (**c**) in 3-month-old male *Runx1*^*f/f*^ *Col2α1-Cre ER* mice induced by vehicle (control) and tamoxifen (TMX). Bar: 50 μm. **d–f** Quantification of (**a**–**c**). The results are presented as the mean ± SD, *n* = 4, **P < 0.01, ***P < 0.001

To further study the role of Runx1 in articular cartilage, we detected cartilage destruction-related gene expression at the protein level. We found that after TMX treatment, the *Runx1*^*f/f*^*Col2α1-Cre ER* mice exhibited significant cartilage erosion with a distinct decrease in Col2α1 expression and a remarkable increase in Mmp13 expression in knee joint cartilage, which reflected sclerosis of cartilage (Fig. 3a, b, d, e). In addition, the cartilage degradation marker Adamts5 was significantly increased in the postnatal Runx1-deficient mice (Fig. 3c, f). These results indicated that the postnatal Runx1-deficient mice exhibited articular cartilage destruction with decreased Col2α1 expression and increased expression of cartilage degradation markers.

### RNA-sequencing analysis showed that Runx1 protected against cartilage loss in OA by regulating the TGFβ, Wnt and Hippo signaling pathways

**Fig. 4:**
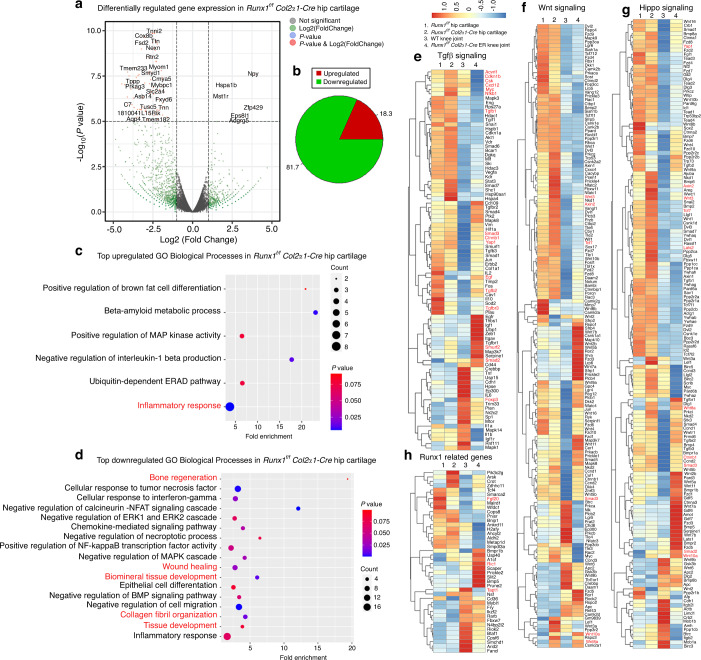
RNA-sequencing analysis of Runx1-deficient hip and knee cartilage showed that Runx1 protected against cartilage loss in OA by regulating the TGFβ, Wnt and Hippo signaling pathways. **a** Volcano plot illustrating differentially regulated gene expression from RNA-seq analysis between the control hip cartilage and cartilage from the *Runx1*^*f/f*^ *Col2α1-Cre* mice. **b** Pie chart of differentially regulated gene expression in hip cartilage. The percentages of genes upregulated and downregulated are shown in red and green, respectively. **c** Gene Ontology (GO) functional clustering of the top enhanced biological process (BP) in *Runx1*^*f/f*^ *Col2α1-Cre* mouse hip cartilage. **d** GO functional clustering of the top decreased BP in *Runx1*^*f/f*^ *Col2α1-Cre* mouse hip cartilage. **e** Heatmap for TGFβ signaling-related gene expression in (1) hip cartilage of the *Runx1*^*f/f*^ mice, (2) hip cartilage of the *Runx1*^*f/f*^ *Col2α1-Cre* mice, (3) knee joint of the WT mice, and (4) knee joint of the *Runx1*^*f/f*^ *Col2α1-Cre ER* mice treated with TMX. **f** Heatmap for Wnt signaling-related gene expression. **g** Heatmap of Hippo signaling-related gene expression. **h** Heatmap for Runx1-related genes

Runx1 is essential for the regulation of transcriptional activities.^7^ To further probe the mechanism underlying the role of Runx1 in OA, we performed RNA-sequencing analysis to explore the Runx1 downstream target genes and related signaling pathways. Differentially expressed genes (DEGs) were found in hip cartilage from the *Runx1*^*f/f*^*Col2α1-Cre* mice and in knee joint tissues of the *Runx1*^*f/f*^*Col2α1-Cre ER* mice treated with TMX (Fig. 4a, Fig. S3A). Volcano plot results showed that the expression of many genes (Fsd2, Ttn, Tnni2, Cox8b, Cmya5, Myom1, and Smyd1) was significantly decreased, Nyp, Hspa1b, Mst1r, Zfp429, Eps8l1, and Adgrg5 levels were markedly increased in the Runx1 conditional knockout hip cartilage (Fig. 4a), while volcano plot results also showed that the expression of many genes (Ear1, Mcpt8, Nxpe2, Ear2, Rab44, Prg3, Hist1h2ag, Mmp25, Retnlg, Atp1a3, Lcn2, Gm30948, Ache and Nbeal2) was significantly decreased, and Prtg, Ptgs2, Al593442, Pthlh and Tnfrsf11b levels were markedly increased in the knee joints from the mice with postnatal Runx1 inducible deletion (Fig. S3A). In the hip cartilage samples, we found 81.7% downregulated genes and 18.3% upregulated genes among the significantly changed genes (Fig. 4b), and in the knee joint samples, we found 68.8% downregulated genes and 31.2% upregulated genes among the significantly changed genes (Fig. S3B). Evaluation of the significant Gene Ontology (GO) BP showed an enhanced inflammatory response and decreased collagen fibril organization, bone regeneration, wound healing, biomineral tissue development, and tissue development (Fig. 4c, d).

Furthermore, we examined the gene expression profiles associated with the Hippo, TGFβ, and Wnt signaling pathways (Fig. 4e–g). Our results showed that many genes were downregulated in the Runx1-deficient hip cartilage, such as Tgfβ2, Tgfbr3, Egf and Foxp3 (Fig. 4e). Tgfβ3 and Egf affect the osteochondrogenic potential of chondrocytes.^14^ TGF-β signaling directly regulates the expression of Foxp3, which contains enhancer elements that allow Smad3 to bind to DNA sequences.^15^ Furthermore, our results showed that some genes upregulated in the Runx1-deficient hip cartilage and knee joints included the TGFβ signaling pathway repressor Smurf2^16^ (Fig. 4e). In addition, we found that Acvrl1, Cdkn1b, Csk, Cxcl12, Myc, Nfkβ and Tgfβ1 were downregulated in the Runx1-deficient knee joints (Fig. 4e). These results suggested that loss of Runx1 could inhibit the expression of some genes in the TGFβ signaling pathway. Moreover, genes associated with activation of the Wnt signaling pathway, such as Wnt8a, Wnt10a, Wnt3, and Axin2,^17^ were upregulated, and genes associated with Hippo signaling were also upregulated, suggesting that these crosslinked signals were also closely related to Runx1 (Fig. 4f, g). Wnt3, Tcf7 and Axin2 were also increased in the Runx1-deficient mice, indicating that loss of Runx1 could promote cartilage ossification and osteophyte formation by activating the Wnt pathway in bone formation (Fig. 4f). Lats2 is an essential component of the Hippo pathway that phosphorylates and inactivates YAP, which is a key link in the activation and shutdown of the Hippo signaling pathway.^18^ Here, we found that Lats2 expression was enhanced in the Runx1-deficient hip cartilage and knee joints, which indicated that Runx1 deficiency in cartilage affected Hippo signaling (Fig. 4g). Chondrocyte-related genes were also downregulated in the hip cartilage of the *Runx1*^*f/f*^*Col2α1-Cre* mice (Fig. S3C). Zhou et al. recently reported a series of genes that interact with Runx1 in chondrocytes.^19^ Examination of the expression profiles of these genes showed altered expression between mutant and WT samples, with differential expression patterns between hip cartilage samples and knee joint samples (Fig. 4h). Runx1 was recently reported to bind the promoters of Tapt1, Fgf20, and Ric1 to upregulate their expression.^19^ Our RNA-seq results demonstrated upregulated Tapt1 expression, downregulated Fgf20 expression, and no significant difference in Ric1 expression in the Runx1-deficient hip cartilage compared to the controls (Fig. 4h). Discrepant results between our study could be due to different materials and methods of Runx1 deletion between the two studies: our study uses in vivo cartilage harvested from mice with chondrocyte-specific deletion of Runx1 using *Col2α1-Cre*, while the study by Zhou et al. utilized si-Runx1 in isolated chondrocyte and culture samples to silence Runx1. RNA-sequencing analysis using knee joints from the *Runx1*^*f/f*^ *Col2α1-Cre ER* mice and the wild-type control mice demonstrated that Runx1’s role as a central regulator in the Hippo/Yap, TGFβ/Smad, and Wnt/β-catenin signaling pathways in articular cartilage differed in knee joint samples compared to hip cartilage alone, indicating that Runx1 regulation is tissue-specific. Collectively, our data are the first to demonstrate that loss of Runx1 may control downstream gene expression by orchestrating the TGFβ, Hippo, and Wnt signaling pathways, thereby exacerbating a series of osteoarthritic pathological processes, including cartilage damage and inflammation.

### Loss of Runx1 in cartilage led to decreased YAP and p-Smad2/3 and increased active β-catenin

**Fig. 5:**
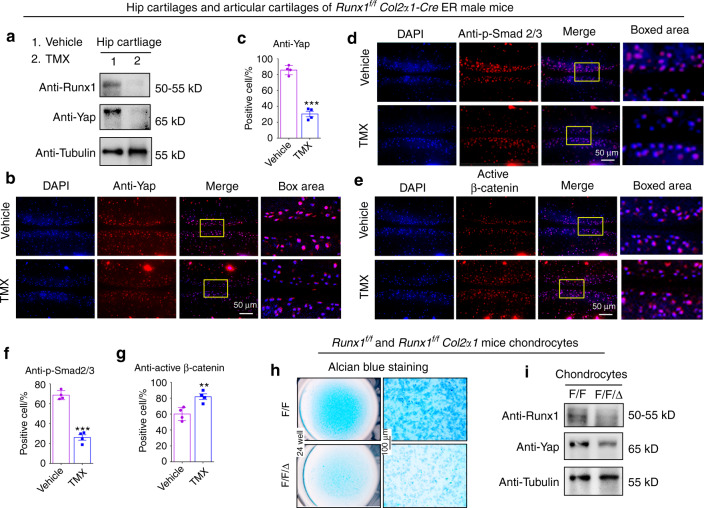
Loss of Runx1 in cartilage led to a decrease in Yap and p-Smad2/3 and an increase in active β-catenin. **a** Western blot of hip cartilage from 2-month-old male *Runx1*^*f/f*^ *Col2α1-Cre ER* mice induced by vehicle (control) or tamoxifen (TMX). **b** IF staining with anti-Yap in 3-month-old male *Runx1*^*f/f*^ *Col2α1-Cre ER* mice. Bar: 50 μm. **c** Quantification of (**b**). **d**, **e** IF staining with anti-p-Smad2/3 and anti-active β-catenin. Bar: 50 μm. **f**, **g** Quantification of (**d** and **e**). **h** Alcian blue staining of primary chondrocytes cultured from newborn *Runx1*^*f/f*^ (F/F) and *Runx1*^*f/f*^ *Col2α1-Cre* (F/F/Δ) mice in chondrogenic medium for 14 days. Bar: 100 μm. **i** Western blot. Primary chondrocytes were induced 14 days from newborn mice. The results are presented as the mean ± SD, *n* = 4, **P < 0.01, ***P < 0.001

Signal integration could provide a better understanding of complex disease processes, and parts of these Hippo/Yap, TGFβ/Smad2/3 and Wnt/β-catenin pathways converge into a complex network that may coregulate transcriptional activators.^20–22^ Furthermore, the expression of important signaling proteins in these pathways was detected in our study. Here, we detected YAP expression in hip cartilage tissues of 2-month-old *Runx1*^*f/f*^*Col2α1-Cre ER* mice. Western blot analysis showed that YAP decreased in the TMX-treated mice (Fig. 5a). We also found that postnatal Runx1 deficiency significantly decreased YAP protein levels in articular cartilage of the 3-month-old male *Runx1*^*f/f*^*Col2α1-Cre ER* mice by IF staining (Fig. 5b, c). Moreover, IF staining results showed that Runx1 knockdown led to a decrease in p-Smad2/3 expression and β-catenin activation in cartilage of the knee joint (Fig. 5d–g), indicating that Runx1 may regulate the Hippo/Yap, Wnt/β-catenin and TGFβ/Smad2/3 signaling pathways to regulate cartilage loss.

In addition, Runx1 interacts with YAP in various cells,^23–25^ but its moderating effect in chondrocytes is unknown. To further validate the regulation of YAP levels through Runx1, we performed experiments in primary chondrocytes from the *Runx1*^*f/f*^ (F/F) and *Runx1*^*f/f*^ *Col2α1-Cre* (F/F/Δ) mice. The results of Alcian blue staining and western blotting showed that Runx1 deletion inhibited chondrocyte matrix deposition with decreased YAP protein levels (Fig. 5h, i). The data indicated that Runx1 may be involved in cartilage regeneration by regulating YAP levels.

### Runx1 overexpression in chondrocytes promoted YAP protein levels in vitro, and local Runx1 overexpression by AAV protected against OA in an ACLT mouse model in vivo

**Fig. 6:**
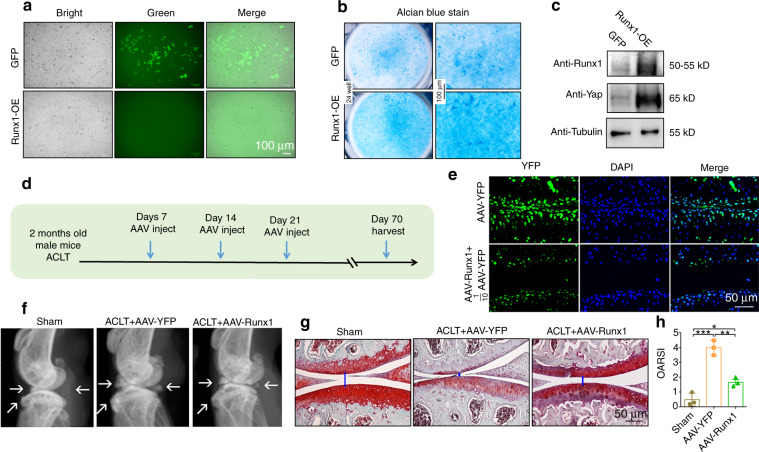
Runx1 overexpression in chondrocytes promoted Yap expression in vitro, and local Runx1 overexpression by AAV protected against OA in an ACLT mouse model in vivo. **a** Images under a fluorescence microscope. pMXs-GFP (GFP) and Runx1 overexpression (Runx1-OE) by pMXs-3xFlag-Runx1 retrovirus transfection in ATDC5 cells that were induced to chondrocytes for 14 days. Bar: 100 μm. **b** Alcian blue staining in ATDC5 cells that were induced to differentiate into chondrocytes for 14 days. Bar: 100 μm. **c** Western blot of induced cells. **d** Schematic display of ACLT surgery-induced OA and subsequent AAV-Runx1/AAV-YFP treatment workflow. **e** Images under a fluorescence microscope for YFP expression in treated mouse knee joints. Bar: 50 μm. **f** Radiography results showed that AAV-Runx1 prevented ACLT-mediated OA. The arrow shows osteophytes and worn articular cartilage. **g** Knee joint safranin O (SO) staining of AAV-treated ACLT-mediated osteoarthritic knees. The blue line shows the space of the knee joint. Bar: 50 μm. **h** OARSI score of (**g**). The results are presented as the mean ± SD, *n* = 3, *P < 0.05, **P < 0.01, ***P < 0.001

To further explore the function and mechanism of Runx1 overexpression in cartilage, we used pMXs-Runx1 and AAV-Runx1 vectors to enhance Runx1 expression in vitro and in vivo. We observed GFP expression under a fluorescence microscope, indicating that the transfection was successful (Fig. 6a). Alcian blue staining showed that Runx1 overexpression significantly increased chondrocyte matrix deposition (Fig. 6b). Furthermore, Western blot results confirmed that Runx1 was successfully overexpressed by pMXs-Runx1 retrovirus transfection in ATDC5 cells (Fig. 6c). Moreover, we found that overexpression of Runx1 in ATDC5 cells by pMX-Runx1 retrovirus transfection significantly enhanced YAP protein levels (Fig. 6c). These results indicated that Runx1 may be essential for chondrocyte matrix deposition and cartilage repair by regulating YAP expression.

To further evaluate the function of Runx1 overexpression in OA, we performed ACLT surgery in 8-week-old WT mice administered AAV-YFP as a control or AAV-Runx1 (Fig. 6d) by intra-articular injection. The articular cartilage surface showed YFP expression, indicating successful AAV infiltration (Fig. 6e). AAV-Runx1 induction was further confirmed by anti-Runx1 immunohistochemistry staining (Fig. S1C). The AAV-Runx1-treated mice were protected from ACLT-induced OA damage compared with the controls (Fig. 6f–h). Moreover, we further detected severe articular cartilage loss in the AAV-YFP-treated osteoarthritic mouse knees with degraded articular cartilage and osteophytes, while the AAV-Runx1 treatment group displayed attenuated articular cartilage damage and recovered knee joint space (Fig. 6f–h). These data suggested that local overexpression of *Runx1* could be an effective target for OA treatment.

## Discussion

**Fig. 7:**
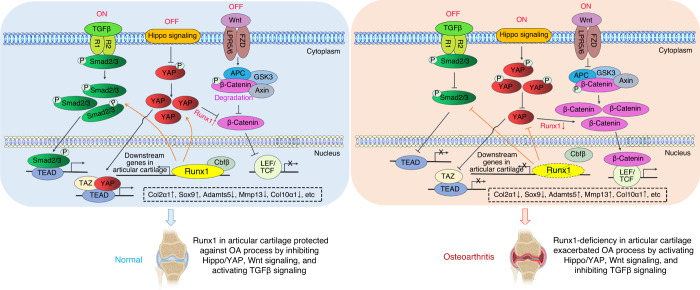
Working model of the role of Runx1 in articular cartilage formation and OA by orchestrating YAP, TGFβ and Wnt signaling

In this study, we found that chondrocyte-specific *Runx1-*deficient mice (*Runx1*^*f/f*^*Col2α1-Cre* and *Runx1*^*f/f*^*Col2α1-Cre ER* mice) developed a spontaneous OA-like phenotype characterized by articular cartilage degradation, osteophyte formation and narrowed joint space. Moreover, AAV-mediated local Runx1 overexpression protected against surgical OA in mice that underwent ACLT. In addition, our data notably indicated that Runx1 could attenuate OA by regulating YAP protein levels and orchestrating multiple signaling pathways, including the Wnt, Hippo, and TGFβ pathways (Fig. 7). Therefore, targeting Runx1 could be an effective therapeutic approach for OA treatment.

Runx1 is a pivotal transcription factor in regulating bone homeostasis and multiple physiological processes. Importantly, our previous work demonstrated that Runx1 is essential for osteoblast differentiation and cartilage development.^8,10^ Genome-wide association studies using clinical samples have found that Runx1 is associated with hip OA and bone fractures.^12^ Fumiko Yano et al. used 2-month-old *Runx1*^*f/f*^*Col2α1-Cre* mice and found that Runx1 could enhance articular cartilage maintenance by increasing cartilage matrix production and decreasing hypertrophic differentiation, but they did not find a significant difference in the 4-month-old *Runx1*^*f/f*^ and *Runx1*^*f/f*^*Col2α1-Cre* mice.^11^ However, their study also found that Col2α1 expression and other chondrogenic markers were significantly decreased in the 4-month-old *Runx1*^*f/f*^ *Col2α1-Cre* mice, which is also in line with our data from the 4.5-month-old *Runx1*^*f/f*^*Col2α1-Cre* mice.^11^ We speculated that the effect of different mouse sources on articular cartilage may have subtle differences over time, as others have also confirmed that *Runx1*^*f/f*^*Col2α1-Cre* mice have an osteoarthritic phenotype,^19^ which we further demonstrated in our current study. Current studies have focused on the role of Runx1 in the early chondrogenic stage, yet the function of Runx1 in postnatal cartilage tissue morphology and physiological changes needs to be further explored. Here, we confirmed that chondrocyte-specific Runx1 deficiency in mice resulted in articular cartilage degradation under physiological conditions and exacerbated articular cartilage destruction under osteoarthritic conditions in DMM and ACLT models. Furthermore, to rule out the confounding effect of Runx1’s role in cartilage development during embryonic development, we employed inducible *Runx1*^*f/f*^*Col2α1-Cre ER* mice and investigated the role of postnatal Runx1 in articular cartilage. Here, mice with postnatal Runx1 deletion exhibited a spontaneous OA-like phenotype with decreased Col2α1 expression and increased Mmp13 and Adamts5 expression. In addition, Mmp13 and Adamts5 play key roles in cartilage degradation during the pathological process of OA.^26^ Recently, Zhou et al. showed that these molecules protect against the pathological progression of OA,^19^ which is consistent with our findings.

Since Runx1 is the scaffold for many signaling pathways, signal integration can provide a better understanding of complex disease processes, and parts of the Hippo/Yap, TGFβ/Smad and Wnt/β-catenin signaling pathways converge into a complex network that may coregulate transcriptional activators.^9,22,27–29^ It has been reported that Runx1 has the potential to regulate TGFβ/BMP, Wnt and ERK/MAPK in bone and other cell types.^28,30–33^ Through the use of genome-wide RNA sequencing, we further explored the mechanisms underlying Runx1’s role in the pathogenesis of OA, which demonstrated that many genes associated with the TGFβ signaling pathway, such as Tgfb2,^34^ Foxp3,^35^ and Egf, were downregulated in Runx1-deficient hip cartilage, while TGFβ signaling pathway repressors, such as Smurf2,^16^ were upregulated. TGF-β signaling directly regulates the expression of Foxp3, which contains enhancer elements that allow Smad3 to bind to DNA sequences.^15^ Tgfβ3 and Egf also affect the osteochondrogenic potential of chondrocytes.^14^ Notably, RNA-seq results also demonstrated downregulated expression of Bmp7 in Runx1-deficient hip cartilage. BMP7 is chondroprotective in OA,^36–39^ and only a narrow range of bioactive TGFβ levels can precisely maintain articular cartilage health.^27^ In addition, phosphorylation of Smad2/3 proteins is a critical step in the TGFβ signaling pathway.^29^ We further examined whether postnatal Runx1 deletion led to decreased p-Smad2/3 expression in the cartilage of the knee joint, suggesting that Runx1 may weaken OA by enhancing the TGFβ signaling pathway. As shown by the RNA-seq data, Yap1, Smad2, Smad3, and CTNNB1 expression was increased in the hip cartilage and knee joint tissue of the *Runx1*^*f/f*^ *Col2α1-Cre* ER mice. Since activation of beta-catenin and phosphorylation of Smad2/3 is a post-translational modification at the protein level, we were not able to detect active beta-catenin and p-Smad2/3 directly through RNA-seq, which explains why the protein level and mRNA level results can be different. Moreover, Runx1 binds Cbfβ to coordinate BMP signaling and Wnt/β-catenin signaling to promote bone formation and inhibit adipogenesis to maintain bone development, but the regulatory mechanism in OA is unclear.^8,9^ Here, the Wnt signaling pathway was enhanced by Runx1 conditional knockout, as shown by our RNA-seq analysis results showing that Wnt8a, Wnt10a, Wnt3, and Ctnnb1 expression was increased in the Runx1-deficient hip cartilage. Ctnnb1 encodes the β-catenin protein, and β-catenin is a pivotal biomarker for detection of Wnt signaling pathway activation.^40^ In addition, Tcf7, Nkd1 and Axin2, as key components in Wnt signaling, were increased in the Runx1-deficient mice. These results indicated that loss of Runx1 could promote cartilage ossification and osteophyte formation by activating the Wnt pathway in bone formation. The Hippo signaling pathway plays an important role in organ size regulation, cell proliferation-differentiation-senescence, carcinogenesis, tissue regeneration, and stem cell function.^41^ Lats2 is an essential component of the Hippo pathway that phosphorylates and inactivates YAP, which is a key link in the activation and shutdown of the Hippo signaling pathway.^18^ Here, we found that Lats2 mRNA expression was increased, and YAP protein expression was decreased in the Runx1-deficient hip cartilage and knee joints. The high expression of Lats2 promoted the phosphorylation of YAP, thereby inhibiting the entry of YAP into the nucleus in Runx1-deficient cartilage, which indicated that Runx1 deficiency in cartilage affected Hippo signaling. Moreover, these results suggested that the loss of Runx1 in cartilage may lead to an increase in intracellular phosphorylated YAP and inhibit the dephosphorylation of YAP into the nucleus. Furthermore, this process would intensify the depolymerization of Axin, APC, GSK3 and the phosphorylated β-catenin complex in the Wnt signaling pathway, thereby releasing more β-catenin into the nucleus and promoting the expression of target genes such as Adamts5, Mmp13 and Col10ɑ1, accelerating cartilage degradation and leading to the occurrence of OA. Notably, the Wnt/β-catenin signaling pathway could promote bone formation in bone metabolism, and the increase in activated β-catenin by the loss of Runx1 in the nucleus may promote osteoblast-related gene expression, also leading to cartilage ossification and osteophyte formation in knee joints. This finding also implied that the regulation of the Wnt/β-catenin pathway by Runx1 may be different in bone formation and osteoarthritic cartilage defects; thus, the mechanism may need to be further explored. In addition, Runx1 deficiency may inhibit the entry of YAP and phosphorylated Smad2/3 into the nucleus, and their interaction with Runx1 in the nucleus will be further weakened, thereby inhibiting the expression of target genes such as Col2ɑ1 and Sox9 and exacerbating the loss of cartilage. Notably, our RNA-sequencing analysis of mutant knee joints showed that Runx1’s role in signaling pathways in articular cartilage is different from that in whole knee joints, indicating that Runx1 regulation is tissue-specific. Collectively, our data first indicated that Runx1 could orchestrate multiple signaling pathways involved in various BP and signaling pathways critical to cartilage regeneration and repair, including the TGFβ, Hippo, and Wnt signaling pathways (Fig. 7).

Zhou et al. recently reported a series of genes that interact with Runx1 in chondrocytes.^19^ Examination of the expression profiles of these genes showed altered expression between mutant and control samples, with differential expression patterns between hip cartilage samples and knee joint samples. Runx1 was recently reported to bind the promoters of Tapt1, Fgf20, and Ric1 to upregulate their expression.^19^ Our RNA-seq results demonstrated upregulated Tapt1 expression, downregulated Fgf20 expression, and no significant difference in Ric1 expression in the Runx1-deficient hip cartilage compared to the controls. Discrepant results between our study could be due to different materials and methods of Runx1 deletion between the two studies: our study used in vivo cartilage harvested from mice with chondrocyte-specific deletion of Runx1 using *Col2α1-Cre*, while the study by Zhou et al. utilized si-Runx1 in chondrocyte isolation and culture samples to silence Runx1. In addition, we found that the expression changes of some genes were not completely consistent in the hip cartilage of the *Runx1*^*f/f*^ *Col2α1-Cre* mice and the knee joints of the *Runx1*^*f/f*^ *Col2α1-Cre ER* with TMX mice, and these specific differences need to be further explored. Both studies by our group and Zhou et al. revealed an important protective function of Runx1 in osteoarthritic cartilage. Collectively, our data indicated that loss of Runx1 may control downstream gene expression by orchestrating the TGFβ, Hippo, and Wnt signaling pathways, thereby exacerbating a series of osteoarthritic pathological processes, including cartilage damage, cartilage ossification and osteophyte formation and inflammation.

YAP is an important signaling molecule in the Hippo and other signaling pathways that regulates cartilage maintenance and the inflammatory response in OA.^42^ YAP activity is not necessary for normal tissue growth and homeostasis, but it plays an important role in tissue regeneration after tissue injury.^43^ It has been reported that Runx1 interacts with YAP in a variety of developmental, hematopoietic stem cell formation, tumorigenesis, and immunotherapeutic processes.^23–25^ YAP also plays an essential role in regulating the Wnt/β-catenin canonical pathway and the noncanonical Wnt pathway^44–46^ implicated in OA.^40,47^ However, the exact function and underlying mechanism of YAP in maintaining cartilage and bone homeostasis is controversial,^44^ and further study is needed to uncover its role in the pathogenesis of OA. Our study showed that Runx1 could control YAP expression in chondrocytes and cartilage in vitro and in vivo.

In addition, OA is a complex disease involving multiple tissues, such as cartilage, bone, muscle, fat and fibroblasts.^1^ The occurrence of OA is also accompanied by a continuous increase in inflammation. Cartilage degrades under inflammation, and subchondral bone gradually erodes cartilage tissue from below through angiogenesis and diffusion of degrading enzymes such as matrix metalloproteinase MMPs.^26^ OA can activate abnormal signaling pathways, such as the NF-κB signaling pathway, resulting in the expression of a large number of catabolic factors and inflammatory mediators. Here, RNA-seq analysis showed that Runx1 deficiency enhanced GO terms of *Runx1*^*f/f*^*Col2α1-Cre* mice, such as positive regulation of the inflammatory response, indicating that Runx1 may suppress the occurrence of inflammation and can be considered a notable target to relieve joint pain in the pathological process of OA.

Our data also demonstrated that AAV-mediated local Runx1 overexpression protected against surgical OA in mice. Targeting Runx1, a regulator of key signaling pathways involved in OA pathogenesis, could facilitate the design of safer and novel therapeutic approaches for OA. However, a limitation of our study is the lack of in-depth mechanisms for Runx1 overexpression in OA. We found that Runx1 overexpression promoted YAP expression in vitro; however, the specific regulatory mechanism of Runx1 overexpression on YAP signaling was still unclear in our in vivo studies. Whether the protective effect of Runx1 overexpression against articular cartilage damage in OA occurs through YAP signaling warrants further investigation in future studies. Our study notably revealed that Runx1 is a key transcription factor in articular cartilage homeostasis and promotes articular cartilage regeneration and repair in OA by orchestrating YAP, TGFβ, and Wnt signaling. Taken together, this work provides important insights into the role of Runx1 in OA and the mechanisms underlying how Runx1 maintains articular cartilage homeostasis. The insights obtained from this study may benefit the development of novel therapeutic approaches for OA.

## Materials and methods

### Generation of chondrocyte-specific Runx1-deficient mice

The *Runx1*^*f/f*^ mouse line was purchased from The Jackson Laboratory. The *Col2ɑ1-Cre ER* mouse line was kindly provided by Professor Di Chen.^48^ The *Col2ɑ1-Cre* mouse line was generously provided by Professor Rosa Serra (University of Alabama at Birmingham, UAB). *Runx1*^*f/f*^ mice were crossed with *Col2ɑ1-Cre* or *Col2ɑ1-Cre ER* mice to obtain *Runx1*^*f/f*^ *Col2ɑ1-Cre* or *Runx1*^*f/f*^ *Col2ɑ1-Cre ER* mice, respectively. Induction of Runx1 deletion was achieved by intraperitoneal (I.P.) injection of TMX or corn oil as a vehicle control as described.^49^ Briefly, TMX (T5648, Sigma) was dissolved in corn oil (C8267, Sigma) at a concentration of 10 mg·mL^−1^ and vortexed until clear. The solution was aliquoted and stored at 4 °C in the dark. Before use, the TMX solution was warmed to room temperature. Three-week-old *Runx1*^*f/f*^ *Col2ɑ1-Cre ER* mice received either TMX or vehicle by I.P. injection continuously for 5 days. The genotypes of the mice were determined by PCR, and the primer sequences were as previously described.^8–10^ All mice were maintained under a 12 h light–dark cycle with ad libitum access to regular food and water at the UAB Animal Facility. Both male and female mice of each strain were randomly divided into groups of five animals each. The investigators were not blinded during allocation, animal handling, or endpoint measurements. The study was approved by the UAB Institutional Animal Care and Use Committee, complied with the National Institutes of Health (NIH) guidelines, and followed all ARRIVE recommendations (Animal Studies: Reporting of In Vivo Experiments) guidelines.

### OA mouse model and AAV-Runx1 treatment

The OA surgical model of DMM was established in *Runx1*^*f/f*^ and *Runx1*^*f/f*^ *Col2α1-Cre* mice. We also used the anterior cruciate ligament transection (ACLT) model to explore the therapeutic effect of Runx1 overexpression on OA. The Osteoarthritis Research Society International score (OARSI) was used to assess cartilage degeneration as previously described.^50^ The workflow of ACLT surgical OA and subsequent AAV-Runx1/AAV-YFP treatment is shown in Fig. 6d. ACLT-treated mice were locally injected with 10 μL of AAV-YFP or AAV-Runx1 (titer > 10^10^ per mL) three times on Day 7, Day 14, and Day 21 in the knee joint cavity and euthanized 70 days after surgery to obtain ACLT knee joint samples.

### Histology and tissue preparation

Histology and tissue preparation were performed as described previously.^10^ Briefly, mice were euthanized, skinned and fixed in 4% paraformaldehyde overnight. The samples were then dehydrated in ethanol solution and decalcified in 10% EDTA for 2–4 weeks. For paraffin sections, samples were dehydrated in ethanol, cleared in xylene, embedded in paraffin, sectioned with a 5 μm Leica microtome and mounted on Superfrost Plus slides (Fisher). For frozen sections, decalcified samples were infiltrated with 30% sucrose, embedded in OCT, and sectioned at 8 μm by a freezing microtome. H&E (HE) staining and safranin O (SO) staining were performed as described previously.^10^ We used a commercial kit (Sigma-Aldrich, 387A-1KT) to perform tartrate-resistant acid phosphatase (TRAP) staining according to the manufacturer’s instructions.

### Radiography

Radioactive images were captured by the Faxitron Model MX-20 at 26 kV by the UAB Small Animal Bone Phenotyping Core associated with the Center for Metabolic Bone Disease.

### Immunohistochemistry and immunofluorescence analysis

Immunohistochemistry (IHC) and immunofluorescence (IF) were performed as previously described.^10^ The following primary antibodies were used: rabbit anti-Runx1 (Abcam, ab23980), mouse anti-Col2α1 (Santa Cruz, sc-52658), rabbit anti-MMP13 (Abcam, ab39012), rabbit anti-ADAMTS5 (Santa Cruz, sc-83186), rabbit anti-Yap (Cell Signaling Technology, 14074 S), p-Smad2/3 (Cell Signaling Technology, 8828 S), and mouse anti-active-β-catenin (Millipore, 05–665). The secondary antibodies were goat anti-rabbit IgG-FITC, goat anti-rabbit IgG-TRITC, goat anti-mouse IgG-FITC and goat anti-mouse IgG-TRITC from Santa Cruz. Images were taken by a Leica DMLB microscope and a Leica D3000 fluorescence microscope. ImageJ software was used to perform counts for the quantification of IHC or IF staining.

### RNA sample preparation and RNA-seq

RNA sequencing and analysis were performed as previously described.^9^ In brief, total mRNA was isolated using TRIzol reagent (Invitrogen Corp., Carlsbad, CA) from mouse knee joint tissue or hip cartilage following the manufacturer’s protocol and was submitted to Admera Health (South Plainsfield, NJ), who assessed sample quality with the Agilent Bioanalyzer and prepared the library using the NEBnext Ultra RNA - Poly-A kit. Libraries were analyzed using Illumina next-generation sequencing, and relative quantification was provided by Admera Health. Read counts were subjected to paired differential expression analysis using the R package DESeq2.^51^

### Western blot

Proteins were loaded on SDS‒PAGE gels and then electrotransferred to nitrocellulose membranes. We used the following primary antibodies: rabbit anti-Runx1 (Abcam, ab23980), rabbit anti-MMP13 (Abcam, ab39012), mouse anti-Sox9 (Santa Cruz, sc-166505), rabbit anti-Col10ɑ1 (Thermo Fisher, PA5-115039), rabbit anti-Yap (Cell Signaling Technology, 14074 S), and mouse anti-β-Tubulin (Santa Cruz, sc-166729). The secondary antibodies were goat anti-rabbit IgG-HRP (sc-2004) and rabbit anti-mouse IgG-HRP (sc-358917) from Santa Cruz.

### Primary chondrocyte culture and ATDC5 cell transfection

We isolated and cultured primary chondrocytes from neonatal *Runx1*^*f/f*^ and *Runx1*^*f/f*^ *Col2ɑ1-Cre* mice as described.^52^ We used pMXs-GFP and pMXs-3xFlag-Runx1 retroviral vectors to package and collect retroviruses, which infected ATDC5 cells to enhance the expression of Runx1. Furthermore, primary mouse chondrocytes and the ATDC5 cell line were induced for 14 days, and Alcian blue staining was carried out to detect chondrocyte matrix deposition as previously described.^10^

### Statistical analysis

The number of animals used in this study was determined in accordance with our previous studies.^8–10^ Experimental data are reported as average ± SD of at least triplicate independent samples. Data were analyzed with a two-tailed unpaired *t*-test. *P* values < 0.05 were considered significant. *P < 0.05, **P < 0.01, ***P < 0.001. Figures are representative of the results.

### Study approval

The study was approved by The UAB Animal Care and Use Committee and conformed to NIH guidelines.

## Supplementary information

Supplemental Figures
