# Supplementary material for: Runx1 is a key regulator of articular cartilage homeostasis by orchestrating YAP, TGFβ, and Wnt signaling in articular cartilage formation and osteoarthritis
Source: Bone Res. 2022 Oct 28;10:63. doi: 10.1038/s41413-022-00231-y (PMC9616925; doi:10.1038/s41413-022-00231-y)
Supplement: Supplementary file 1 — Supplemental Figures [file 41413_2022_231_MOESM1_ESM.docx]

**Runx1 is a key regulator of articular cartilage homeostasis by orchestrating YAP, TGFβ, and Wnt signaling in articular cartilage formation and osteoarthritis**

**Running title:** Function of Runx1 in articular cartilage formation and OA

Yan Zhang^1,2,#^, Tao Zuo^1, 3,#^, Abigail McVicar^4^, Hui-Lin Yang^3^,  Yi-Ping Li^1,4*^, Wei Chen^1,4*^

**Supplemental Figures**


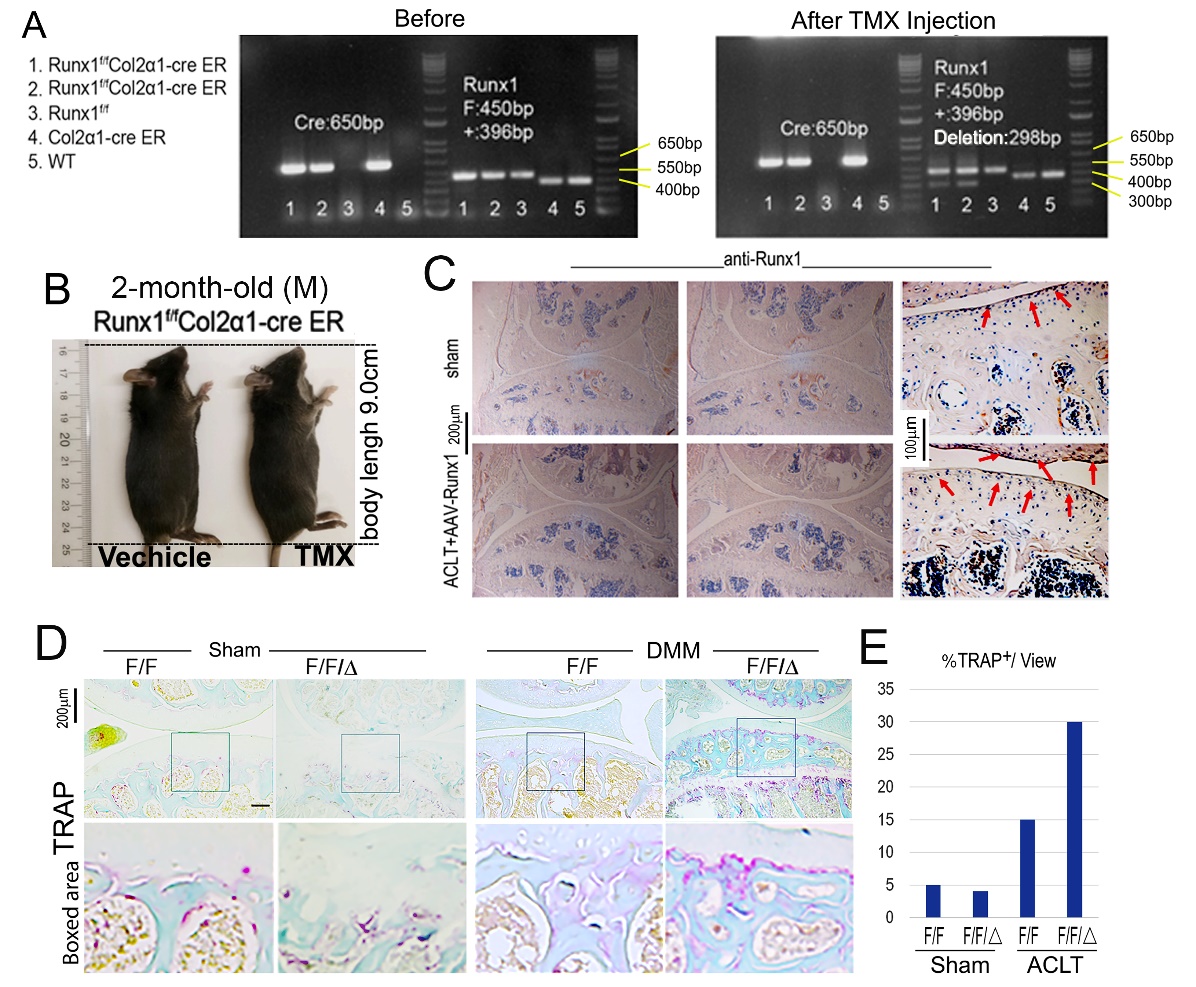


**Supplemental Figure 1. Genotyping and Body lengths of *Runx1^f/f^ Col2α1-Cre ER* mice, TRAP staining for knee joint of Runx1^f/f^ Col2α1-Cre mice. (A)** Gel images of PCR genotyping for *Runx1^f/f^ Col2α1-Cre ER* mice before and after tamoxifen (TMX) induction. **(B)** Body lengths from 2 month-old male *Runx1^f/f^ Col2α1-Cre ER* mice induced by vehicle (control) and TMX. **(C)** Anti-Runx1 immunohistochemistry staining. **(D)** TRAP staining for knee joint of 3-month-old mice after sham or DMM surgery in Runx1^f/f^ Col2α1-Cre mice. Bar: 200 μm and 100 μm. **(E)** Quantification of TRAP^+^ cells in D as % TRAP^+^ cells/view.


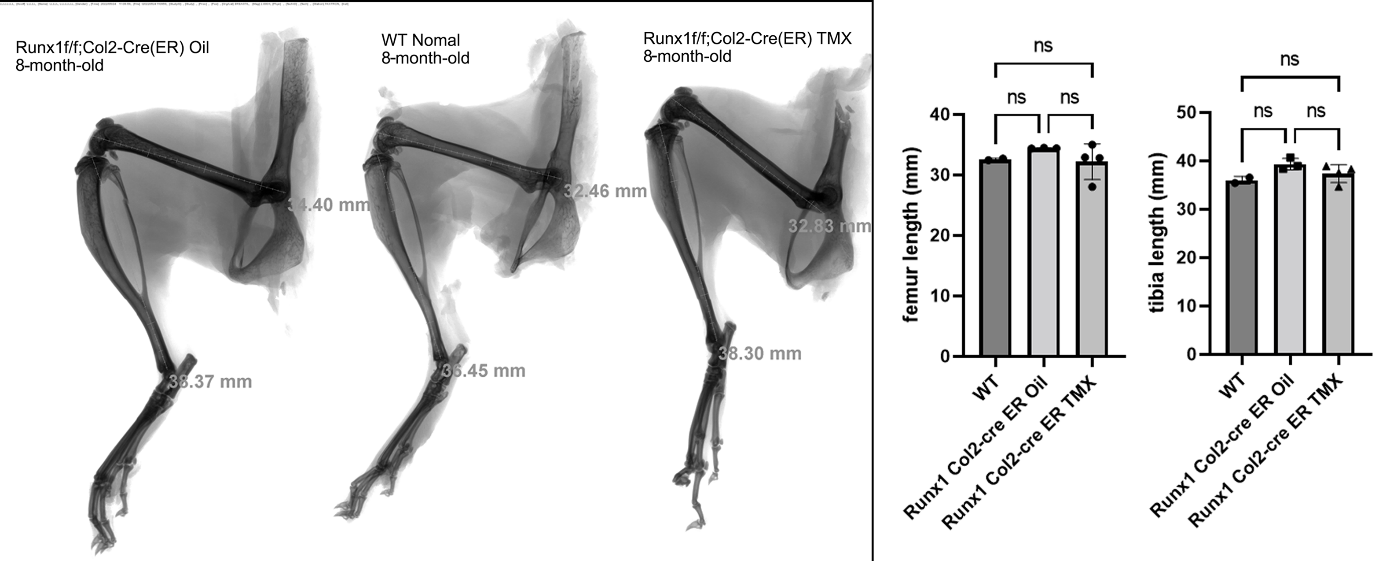


**Supplemental Figure 2. Femur and tibia lengths of *Runx1^f/f^ Col2α1-Cre ER* mice.** X-ray images of 8-month-old WT and *Runx1^f/f^ Col2α1-Cre ER* mice induced by vehicle (Oil) and TMX, and quantification of tibia and femur lengths. Results are presented as mean ± SD; ns, not significant.


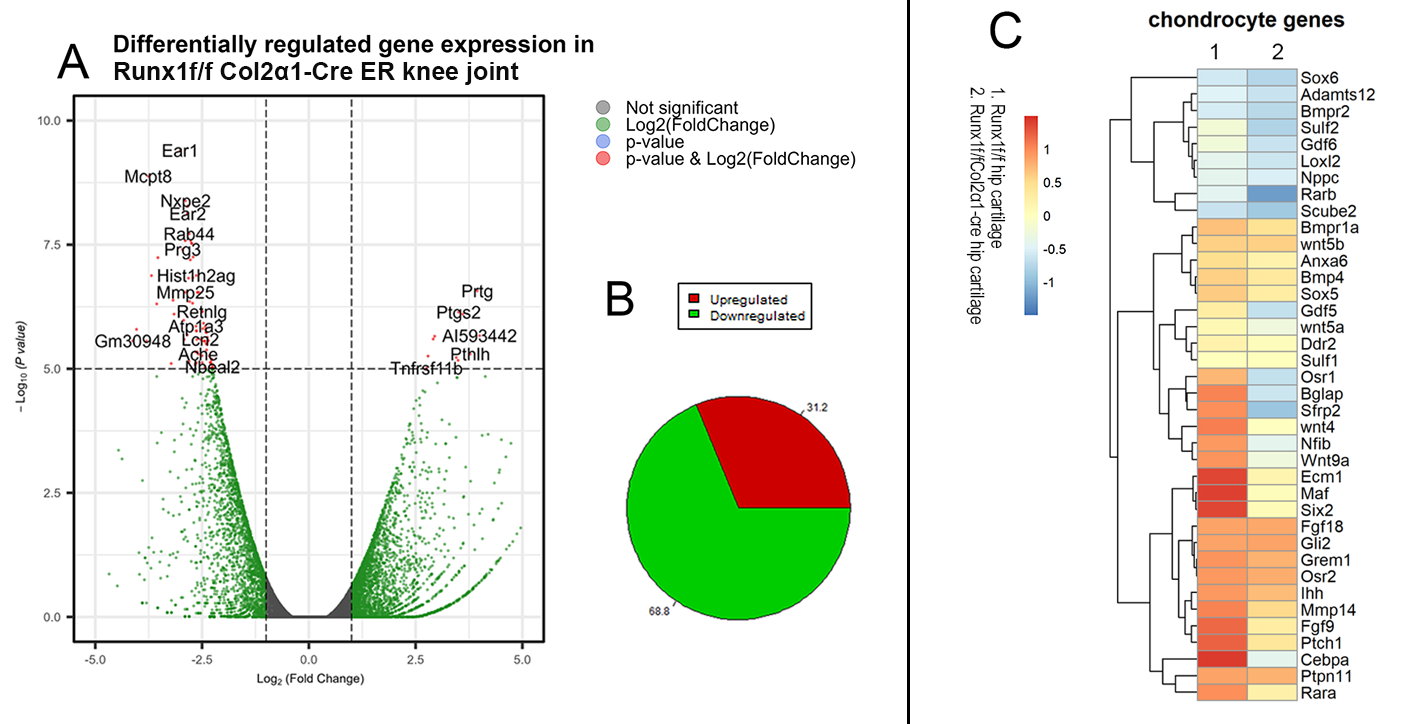


**Supplemental Figure 3. RNA-sequencing analysis showing** **differentially expressed genes in *Runx1^f/f^ Col2α1-Cre* ER mice knee joint, and chondrocyte genes expression in Runx1-deficiency cartilage. (A)** Volcano plot illustrating differentially regulated gene expression in knee joints from RNA-seq analysis between control (WT) and *Runx1^f/f^ Col2α1-Cre ER* mice treated with TMX. **(B)** Pie chart for differentially regulated genes expression in knee joint of *Runx1^f/f^ Col2α1-Cre ER* mice treated with TMX. Percentage of genes upregulated and downregulated are shown in red and green, respectively. **(C)** Heatmap for chondrocyte related genes expression in (1) hip cartilage of *Runx1^f/f^* mice and (2) hip cartilage of *Runx1^f/f^ Col2α1-Cre* mice.
